# Molecular evidence for the evolution of ichnoviruses from ascoviruses by symbiogenesis

**DOI:** 10.1186/1471-2148-8-253

**Published:** 2008-09-18

**Authors:** Yves Bigot, Sylvie Samain, Corinne Augé-Gouillou, Brian A Federici

**Affiliations:** 1 Université François Rabelais de Tours, GICC, UFR des Sciences & Techniques, Parc de Grandmont, 37200 Tours, FRANCE; 2 CNRS, UMR 6239, UFR des Sciences & Techniques, Parc de Grandmont, 37200 Tours, France; 3 CHRU de Tours, Bd Tonnelé, 37032 Tours, France; 4 GENOSCOPE, 2 rue Gaston Crémieux, CP 5706, 91057 Evry, France; 5 Department of Entomology & Graduate Programs in Genetics, Microbiology, and Molecular Biology, University of California, Riverside, CA92521, USA

## Abstract

**Background:**

Female endoparasitic ichneumonid wasps inject virus-like particles into their caterpillar hosts to suppress immunity. These particles are classified as ichnovirus virions and resemble ascovirus virions, which are also transmitted by parasitic wasps and attack caterpillars. Ascoviruses replicate DNA and produce virions. Polydnavirus DNA consists of wasp DNA replicated by the wasp from its genome, which also directs particle synthesis. Structural similarities between ascovirus and ichnovirus particles and the biology of their transmission suggest that ichnoviruses evolved from ascoviruses, although molecular evidence for this hypothesis is lacking.

**Results:**

Here we show that a family of unique pox-D5 NTPase proteins in the *Glypta fumiferanae *ichnovirus are related to three *Diadromus pulchellus *ascovirus proteins encoded by ORFs 90, 91 and 93. A new alignment technique also shows that two proteins from a related ichnovirus are orthologs of other ascovirus virion proteins.

**Conclusion:**

Our results provide molecular evidence supporting the origin of ichnoviruses from ascoviruses by lateral transfer of ascoviral genes into ichneumonid wasp genomes, perhaps the first example of symbiogenesis between large DNA viruses and eukaryotic organisms. We also discuss the limits of this evidence through complementary studies, which revealed that passive lateral transfer of viral genes among polydnaviral, bacterial, and wasp genomes may have occurred repeatedly through an intimate coupling of both recombination and replication of viral genomes during evolution. The impact of passive lateral transfers on evolutionary relationships between polydnaviruses and viruses with large double-stranded genomes is considered in the context of the theory of symbiogenesis.

## Background

Parasitic wasps belonging to the families Ichneumonidae and Braconidae are among the most successful higher eukaryotic organisms. These two large families contain more than 37,000 known species, and estimates of the total number of species are as high as 100,000 [1]. Approximately two-thirds of these wasps are endoparasites, meaning that the larval stages develop within the body cavity of their hosts, typically other insects. Among the most successful of these endoparasitic wasps are those that use lepidopteran larvae as hosts. Owing to the economic importance of these insects and the utility of their wasp parasites as biological control agents, the ability of these parasites to develop within lepidopteran hosts without triggering an intense immune response has been the subject of numerous studies over the past forty years.

Early studies of the Mediterranean flour moth, *Ephestia kuhniella*, parasitized by the ichnemonid, *Venturia canescens*, showed that eggs of this species are coated with particles that resemble virions [2-4] and contain surface proteins that mimic host proteins, thus keeping the eggs and larvae from being recognized as foreign material by their host. These particles lack DNA, and thus are not considered virions [5]. With respect to both species number and mechanisms that lead to successful parasitism, endoparasitic wasps are known to inject secretions at oviposition, but only a few lineages use viruses or virus-like particles (VLPs) to evade or to suppress host defences. In the family Ichneumonidae, for example, four types of host defence suppression mediated by the injection of fluids or suspensions are known that lead to successful parasitism.

1) Fluid injected with eggs bypasses host defences without the aid of viruses or VLPs [6].

2) Wasps inject a virus that replicates in both the wasp and lepidopteran host. One example is the wasp *Diadromus pulchellus*, which injects an ascovirus, DpAV4 [7] into host pupae to circumvent host defence response.

3) The wasp injects VLPs capable of molecular mimicry and/or direct defence suppression.

4) The wasp injects polydnavirus particles that contain genes coding for proteins that interfere with host defence responses.

The last mechanism is by far the best-studied type of direct immune suppression by ichneumonid wasps, and occurs in many species belonging to genera *Campoletis*, *Hyposoter *and *Tranosema *(Ichneumonidae, Campopleginae), and *Glypta *(Ichneumonidae, Banchinae) [8]. In these cases, female wasps inject eggs along with ichnovirus particles into their hosts. Similarly, in certain lineages of endoparasitic braconid wasps, other types of immunosuppressive particles containing DNA occur in the fluid injected along with eggs [[9]; for a review, [10]]. Once in the host, ichneumonid and brachonid particles enter host nuclei and their DNA is transcribed, producing proteins that selectively suppress various steps in the host defence response. As a result of this unusual biology, these particles were described as symbiotic viruses belonging to new viral family, *Polydnaviridae *[10-12]

Since the 1970's, it was assumed that the DNA in the polydnavirus particles, as with all other viruses, encoded typical enzymes and proteins for viral replication and virion assembly and structure. However, several recent genomic studies have shown that only a small number of the genes vectored into lepidopteran hosts, less than 2%, have homologs in other viruses. Most viral DNA is non-coding, except that which codes for wasp proteins involved in suppression of immune pathways, such as phenoloxidase activation and the toll pathways [8,13,14]. Even before these genomic studies, it was suggested that these particles were more similar to organelles than viruses [15]. The similarities between particle structure and virions of known types of complex DNA insect viruses are striking, and suggest these immunosuppressive particles originated by symbiogenesis between viruses and endoparasitic wasps, the same evolutionary process by which mitochondria and plastids originated from symbiotic bacteria [16]. For example, most braconid wasps produce enveloped bacilliform particles classified as bracoviruses, and these resemble baculovirus and nudivirus virions [10,15]. Similarly, ichneumonid wasps produce enveloped spindle-shaped particles classified as ichnoviruses that resemble virions of ascoviruses, viruses lethal to lepidopterans, which, interestingly, are vectored by endoparasitic wasps [15]. It must also be noted that ichnoviruses resemble other true virus particles that are structurally very similar to virions of ascoviruses, but which remain unclassified because the lack of information about their genomes [17-21]. However, ascoviruses and ichnoviruses display very different genome properties; similar genomic differences occur between bracoviruses and baculoviruses or nudiviruses, suggesting that convergent evolution led to the origin the different polydnavirus types from at least two different types of viruses.

In ascoviruses, the genome consists of a single circular DNA molecule ranging from 119- to 180-kpb in size [7]. Phylogenetic analyses of several viral genes have revealed that ascoviruses are closely related to iridoviruses [22], and likely evolved from them. In contrast, the genome of ichnoviruses is composed of multiple circular DNA molecules (25 to 105) representing a total size of 250 to 300-kbp, all of which are replicated from the wasp chromosomes. The ichnovirus proviral genome is specifically excised and amplified in several segments in the female calyx cells, the only wasp tissue in which ichnovirus virogenesis occurs. After assembly, these particles are secreted into the female genital tract. Once injected into the host, the ichnovirus genome does not replicate, and does not lead to the production of a new virus generation. The third characteristic of ichnoviruses is that most of the genes borne by the particles are not related to viral genes. Among the 7 annotated ichnovirus gene families, there are four (rep, PRRP, N, and TrV) for which no homology with known eukaryotic (or prokaryotic) proteins has been detected and for which no function has been proposed. Among the remaining three (cys, ank and inx), cys-motif proteins have no clear homologs among eukaryotic (or prokaryotic) proteins, although the "cysteine knot" that they form is a folding domain found in many proteins, but not one that is necessarily related to eukaryotic host immune systems [10,14]. However, some protein domains and their putative functions suggest that they might be related to regulatory components of eukaryotic host defence systems that are not sufficiently elucidated.

Although the resemblance of the polydnavirus virions to those of conventional insect viruses suggests that the former evolved from the latter, to date no molecular evidence supports this hypothesis. In the case of ascoviruses and ichnoviruses, well-conserved genes found among the three ascoviruses sequenced so far (SfAV1a [23], TnAV2c [24], and HvAV3e [25]) are not found in ichnovirus genomes. As noted above, the principal reason for this is that the genomes of the latter viruses appear to contain mainly wasp genes, not viral genes. This highlights the need for new and alternative types of sequence data obtained from pertinent biological systems. In this regard, DpAV4 has features that could provide important insights. Indeed, it is the only ascovirus known to replicate in both its wasp and caterpillar hosts. It is transmitted vertically from wasp to caterpillars to suppress the defence response of the latter host, thereby enabling parasite development [26,27]. Moreover, in males and females of *D. pulchellus*, the DpAV4 genome resides in the nuclei of all hosts cells, providing a possible example of what may have been an intermediate stage in the symbiogenesis that led to the evolutionary origin of ichnoviruses.

We recently sequenced the DpAV4 genome, and a combination of our analysis of this genome and recent data from new types of ichnoviruses, as well as new software programs that elucidate protein relationships based on structural analysis, have enabled us to detect phylogenetic relationships between proteins encoded by open reading frames of DpAV4 and the *Glypta fumiferanae *(GfIV) and *Campolitis sonorensis *(CsIV) ichnoviruses. In support of the symbiogenesis hypothesis for the origin of ichnoviruses, data and analyses suggest two independent symbiogenic events, in agreement with what was previously proposed [28]. The first led to the ichnoviruses in Banchinae lineage. This hypothesis is based on the occurrence of a gene cluster present in GfIV and DpAV4. The second symbiogenic event led to ichnoviruses in the Campopleginae wasp lineage. This hypothesis is based on relationships of the major capsid proteins among CsIV, ascoviruses and iridoviruses. Extending our investigations to proteins encoded by open reading frames of certain ascoviruses and bracoviruses, hosts and bacteria, in the light of recent analyses about the involvement of the replication machinery of virus groups related to ascoviruses in lateral gene transfer [29], we discuss the robustness and the limits of the molecular evidence supporting an ascovirus origin for ichnovirus lineages.

## Results and discussion

### DpAV4 and ichnovirus related proteins

**Figure 1:**
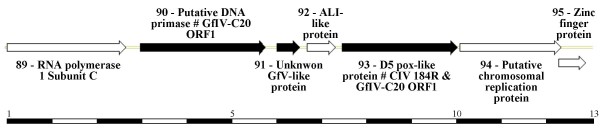
**Map of the 13-kbp region of the DpAV4 genome (EMBL Acc. N°**CU469068**and**CU467486) **that contains the gene cluster with direct homologs in the genome of the *Glypta fumiferanae *ichnovirus.** DpAV-4 ORF with well-characterized direct homologs among other ascovirus and iridovirus genomes are represented by white arrows. Homologous ORF of the GfIV genes are represented by black arrows. The sequence and the features of the protein encoded by these ORF are detailed in fig. S1. Below, the graph is scaled in kbp.

The DpAV4 genome sequenced by Genoscope (France) is 119,334-bp in length. Its organization, gene content and evolutionary characteristics will be detailed in a separate publication (manuscript in preparation; Additional file 1). However, BLAST results obtained with several ORFs in the DpAV4 genome provide evidence that certain ichnovirus ORFs have their closest relatives in an ascovirus genome. Specifically, we identified a 13-kbp region that contains a cluster of three genes (Fig. 1, ORF90, 91 and 93; Additional files 1 and 2) that have close homologs in a GfIV gene family composed of seven members [28]. All contain a domain similar to a conserved domain found in the pox-D5 family of NTPases. To date, this pox-D5 domain has been identified as a NTP binding domain of about 250 amino acid residues found only in viral proteins encoded by poxvirus, iridovirus, ascovirus and mimivirus genomes. These genes seem to be specific to GfIV, as they are absent in the three sequenced genomes of other ichnoviruses, namely CsIV, *Tranosema rostrales *ichnovirus (TrIV), and *Hyposoter fugitivus *ichnovirus (HfIV).

**Figure 2:**
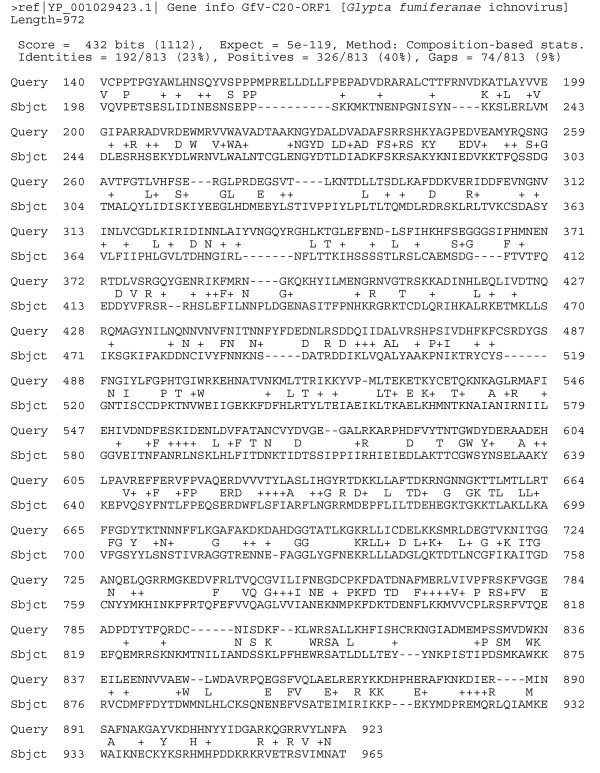
**Amino acid sequence comparison resulting from a BLAST search done with the DpAV4 ORF90 as a query, and the best hit corresponding to the protein encoded by the ORF1 of the ichnovirus segment GfV-C20** (Subject; Genbank Acc. N° YP_001029423).

More specifically, in DpAV4, ORF90 encodes a protein of 925 amino acid residues that is 40% similar from position 140 to 925 to a protein of 972 amino acid residues encoded by the ORF1 contained in the segment C20 in the GfIV genome (Fig. 2). These two proteins can therefore be considered putative orthologs. The 480 C-terminal residues of this DpAV4 protein are also 42% similar to the C-terminal domain of the protein homologs encoded by the ORF1 of the D1 and D4 GfIV segments, 36% similar to the N-terminal and the C-terminal domains of the protein encoded by the ORFs 184R and 128L of the iridovirus CIV and LCDV, and 30% similar with those encoded by ORFs 119, 99 and 78 in the ascovirus genomes of HvAV3e, SfAV1a and TnAV2c, respectively. Overall, this indicates that this DpAV4 protein is more closely related to that of GfIV than to those found in other ascovirus and iridovirus genomes currently available in databases. ORF091 encodes a protein of 161 amino acid residues similar only with the C-terminal domain of three proteins encoded by the ORFs 1, 1 and 3, contained, respectively, in GfIV segments D1, D4 and D3. In contrast, ORF93 is closer to iridovirus and ascovirus genes than to GfIV genes. This protein of 849 amino acid residues is 43% similar over all its length to CIV ORF184R orthologs in all iridoviral and ascoviral genomes and is only 36% similar over 350 amino acid residues to the C-terminal domain of the GfIV protein homologs encoded by the ORF1, 2, 1, 1, 1 and 1 in, respectively, the C20, C21, D1, D2, D3 and D4 segments of this virus.

Analysis of the genes surrounding the DpAV4 ORF-90-91-93 cluster confirms that this virus has an ascovirus origin since this region contains ORFs that are close homologs of genes in iridovirus and ascovirus genomes. Upstream from the ORF-90-91-93 cluster, an ORF encoding the DNA-dependent RNA polymerase 1 subunit C is present, which is an ortholog of the iridoviral CIV ORF176R and the ascoviral SfAV1a ORF008. Downstream from this cluster, there are two genes, absent in known ascoviral genomes, but similar to the iridoviral CIV ORF115L and CIV ORF132L. These two genes encode, respectively, a chromosomal replication initiation protein and zinc finger protein. In between them, a gene encoding a small protein is present that is similar to that encoded by the ORF069L of the iridovirus CIV, and which corresponds to the ALI-like protein also found in entomopoxviruses [30].

Since the three DpAV4 genes have relatives in all ascovirus and iridovirus genomes sequenced so far, their presence in the DpAV4 genome cannot result from a lateral transfer that occurred from an ichnovirus genome related GfIV to DpAV4. Thus, as these DpAV4 genes are the closest relatives of the pox-D5 gene family present in GfIV identified so far, they could be considered a landmark of the symbiogenic ascovirus origin of the ichnovirus lineage to which this polydnavirus belongs. An alternative explanation is that the presence of DpAV4-like genes in the genome of GfIV resulted from a lateral transfer from viral genomes closely related to those of GfIV and DpAV4. Indeed, this might have happened when a *Glypta *wasp was infected by an ancestral virus related to DpAV4. Nevertheless, the symbiogenic origin of GfIV from ascoviruses is also supported by morphological features of its virions [28], which, aside from similarities in shape, also show reticulations on their surface in negatively stained preparations, a characteristic of the virions of all ascovirus species examined to date [7].

### Relationships between ascovirus and ichnovirus virion proteins

**Figure 3:**
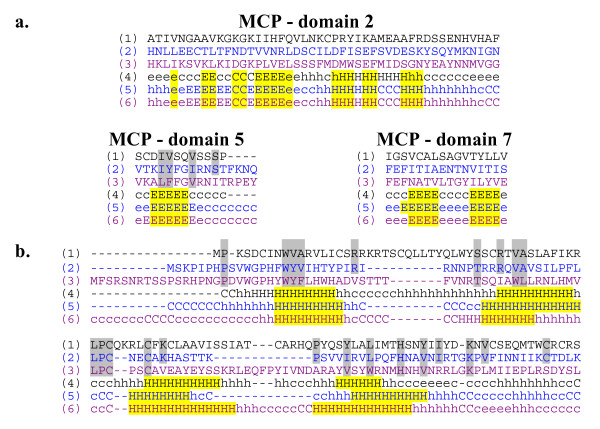
**Sequence (lanes 1 to 3) and secondary structure (lanes 4 to 6) comparisons among (a) MCP and (b) SfAV1a ORF061 orthologs from CsIV (lanes 1 and 4, typed in black), DpAV4 (lanes 2 and 5, typed in blue) and SfAV1a (lanes 3 and 6, typed in purple).** Conserved positions among the amino acid sequence of CsIV and those of DpAV4 and SfAV1a are highlighted in grey. Secondary structures in the three SfAV1a ORF061 orthologs were calculated with the Network Protein Sequence Analysis at http://npsa-pbil.ibcp.fr/ and the statistical relevance of the secondary structures were evaluated with Psipred at http://bioinf.cs.ucl.ac.uk/psipred/. C, E and H in lanes 4 to 6 respectively indicated for each amino acid that it is involved in a coiled, b sheet or a helix structure. Using default parameters of Psipred, upper case letters indicate that the predicted secondary structure is statically significant in Psipred results. Significant secondary structures are highlighted in yellow. In (a), the comparisons were limited to three of the seven conserved domains (Additional file 3a, b and 3c), the 2, 5 and 7. Indeed, classical *in silico *methods appeared to be inappropriate to predict statistically significant secondary structures in conserved structural protein rich in b strand such as iridovirus and ascovirus MCP. In contrast, a complete and coherent domain comparison was obtained by HCA profiles (fig. S3b, c).

Because ascovirus virions and ichnovirus particles display structural similarities, we developed an approach to search for homologs of virion structural proteins in ichnoviruses. These approaches were initiated in 2000 and recently finalized, but some of the conclusions have been published [14]. To date, only two virion proteins from the *Campoletis sonorensis *ichnovirus (CsIV) have been characterized [31,32]. The first is the P44 (Acc N° AAD01199), a structural protein that appears to be located as a layer between the out envelope and nucleocapsid, and the second, P12, a capsid protein (Acc N° AF004367). Presently, there are more than one hundred ascoviral or iridoviral MCP sequences in databases. BLAST searches using these sequences failed to detect any similarities between CsIV virion proteins and ascoviral or iridoviral MCPs, or any other proteins [33]. To evaluate the possibility that homology between ichnovirus and ascovirus virion proteins may simply not be detectable by conventional Blastp searches, we used a different method, WAPAM (weighted automata pattern matching; [34]). The models were designed on the basis of a previous study [22] demonstrating that MCP encoded by ascovirus, iridovirus, phycodnavirus and asfarvirus genomes are related, and all contain 7 conserved domains separated by hinges of very variable size. We investigated these conserved domains further using hydrophobic cluster analysis (HCA, [35]). This analysis revealed that most conservation occurred at the level of hydrophobic residues, as expected for structural proteins (Additional file 3a and 3b). The size variability of the hinges between conserved domains and the conservation of hydrophobic residues might explain why BLAST searches using iridoviral and ascoviral MCP sequences have limited ability to detect MCP orthologs in phycodnavirus and asfarvirus genomes. We designed two syntactic models (see Materials and Methods), which together were able to specifically align all MCP sequences of the four virus families. Importantly, WAPAM aligned the CsIV ichnovirus P44 structural protein with both models. Complementary structural and HCA confirmed the presence of the seven conserved domains in this CsIV structural protein (Fig. 3a and Additional file 3c).

In addition to the above analysis, ten syntactic models were developed using proteins conserved in the three sequenced ascovirus species (SfAV1a, TnAV2c, and HvAV3a) and twelve iridoviruses [36]. None of these models detected homologs among ichnovirus proteins available in databases, except for one (see Materials and Methods section), developed from small proteins encoded by the DpAV4 ORF041, SfAV1a ORF061, HvAV3a ORF74, and TnAV2c ORF118 in the ascovirus genomes, and iridovirus CIV ORF347L and mimivirus MIV ORF096R genomes, respectively. Importantly, these proteins have orthologs in vertebrate iridoviruses, phycodnaviruses, and asfarvirus. In SfAV1a, the peptide encoded by ORF061 is one of the virion components. In ascoviruses, iridoviruses, phycodnaviruses, and the asfarvirus, they have been annotated as thioredoxines, proteins that play a role in initiating viral infection [37-39]. Database mining with our model revealed four hits with CsIV sequences (Acc N°. M80623, S47226, AF236017, AF362508) each a homolog ORF of SfAV1a ORF061. In fact, these sequences correspond to several variants of a single region contained in the B segment of the CsIV genome. To date, these have not been annotated in the final CsIV genome, probably because they overlap a recombination site. HCA analyses confirmed that the hydrophobic cores were conserved (Fig. 3b and Additional file 3d and 3e).

The chromosomal locations of genes encoding these two CsIV proteins, i.e., P44 and P12, were also consistent with the symbiogenesis hypothesis. In fact, the ORF encoding P44 is not found in proviral DNA. It is notable that no ORFs encoding orthologs of P44 or other structural proteins such as MCPs are found in any of the other three ichnovirus genomes sequenced – TrIV, GfIV, HfIV [8,14]. Therefore, this indicates that the orthologs of ichnovirus MCPs and other virion structural proteins are also probably located in the genomes of these wasps, i.e., not in proviral DNA. In contrast to this, we found that the gene encoding the CsIV ortholog of SfAV1a ORF061 is located within the proviral DNA. Whether ortholog proteins are similarly involved in the TrIV, GfIV and HfIV biology, their genes are not found in proviral DNA, since no matches were detected in their viral genomes. The phylogenetic analysis performed previously on P44 and the SfAV1a ORF061 orthologs [15] indicated that they have an ancestor close to that of the ascoviruses and iridoviruses.

As in the case of genes encoding pox-D5 family of NTPases in all ascoviruses, iridoviruses, and GfIV, genes encoding virion proteins cannot result from a horizontal transfer from a Campoplegine or Banchine ichnovirus genome to all ascovirus, iridovirus, phycodnaviruses and asfarvirus genomes. As the ascovirus genes encoding the two virion proteins investigated here are the closest relatives of virion proteins in CsIV, they can be considered a landmark reflecting the symbiogenic origin of the two ichnovirus lineages from ascoviruses closely related to DpAV4. In fact, the difficulty encountered in elucidating their sequence relationships can be explained by a combination of the marked transition from ascovirus to ichnovirus, and the significant selection constraints that resulted as the latter virus type evolved from the former.

### Impact of the intimate environment on lateral gene transfer

Analysis of available ascovirus, iridovirus and ichnovirus genomes provides some of the first molecular support for the hypothesis that ichnoviruses evolved from ascoviruses by symbiogenesis. However, examining genes shared only by ascovirus, iridovirus and ichnovirus genomes likely limits the sources of genes that contributed to the evolution and complexity of these viruses, especially of the role of lateral gene transfer. Relevant to this is the recent finding that an important part of the mimivirus and phycodnavirus genomes had a bacterial origin [28]. Obviously, this did not lead to the conclusion that these viruses had a bacterial origin. The cytoplasmic environment in which these viruses replicate is rich in bacterial DNA because their amobae and unicellular algae hosts feed on bacteria that they digest in their cytoplasm. Thus, it has been proposed [28] that lateral transfers of bacterial DNA within these viral genomes were driven by intimate coupling of recombination and viral genome replication. Indeed, replication of these viruses is similar to that of bacteriophage T4. This mode of replication has been called recombination-primed replication. It permits integration of DNA molecules with sequence homology as short as 12-bp [28,40]. The replication machinery used by ascoviruses, iridoviruses, mimiviruses, phycodnaviruses, and other nucleocytoplasmic large DNA viruses (NCLDV) [41,42] is common to all of them, despite differences in the specifics of replication in each virus family. It can therefore be expected that recombination-primed replication occurred repeatedly during evolution of both these viruses and the genome of their eukaryotic hosts. In an eukaryotic cellular environment in which bacteria, chromosomes, NCLDV viruses and non-NCLDVs (such as baculoviruses) intimately cohabit temporarily or permanently, recombination-primed replication is able to allow reciprocal passive lateral transfers between viral genomes, host chromosomes, and bacterial DNA. Under these conditions, lateral transfers are considered passive since they just result from the intimate environment and not from an active mechanism dedicated to genetic exchanges. In ascoviruses and iridoviruses, the occurrence of such lateral transfers is supported by BLASTp searches that detected the presence of ORFs whose closest relatives have their origin within eukaryotic genomes (e.g., for DpAV4, in Additional data 1, ORFs 029, 049, 077, 080, 083, 118), bacterial genomes (e.g., for DpAV4, in Additional data 1, ORFs 056, 057, 059, 112, 115 and119) or viruses belonging to other NCLDV and non-NCLDV families (e.g., for DpAV4, in Additional data 1, ORFs 007, 037, 062, 068).

**Figure 4:**
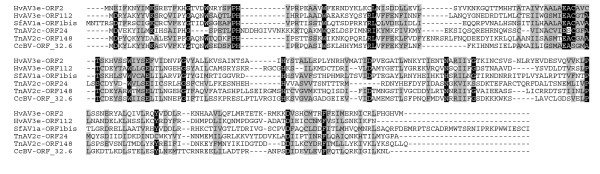
**Amino acid sequence analysis of the N-acetyl-L-glutamate 5-phosphotransferase-like proteins encoded by one gene in SfAV1a (ORF1bis), two genes in HvAV3e (ORFs 002 and 112) and TnAV2c (ORFs 024 and 148), and one gene in CcBV.** Identical residues between sequences are highlighted in black typed in white, those that are similar are highlighted in grey.

The transmission of ascoviruses is unusual in that they are poorly infectious *per os *and appear to be transmitted among lepidopteran hosts by parasite wasp vectors at oviposition [7,43]. The genome of the ascoviruses can be replicated in presence of polydnavirus DNA either within the reproductive tissues of female wasps or within the body of the parasitized hosts infected by both polydnavirus and ascovirus. Consequently, integrated sequences of ascovirus origin can be expected within wasp and polydnavirus genomes. Reciprocally, sequences of polydnavirus origin may have been integrated in ascovirus genomes, whatever the wasp origin, ichneumonid or braconid. One gene family related to a bacterial family of N-acetyl-L-glutamate 5-phosphotransferase (Acc. N° of the closest bacterial relatives YP_001354925, CAM32558, ZP_00944224, ZP_02006449), identified only within the SfAV1a, HvAV3e and TnAV2c genomes, supports this conclusion. It has been found in the genome of a bracovirus, *Cotesia congregata *BracoVirus (CcBV [13]; Fig. 4). Since this gene is absent in the genome of *Microplitis demolitor *BV, a related bracovirus [8], it is difficult to infer the direction of the lateral transfer between the common ancestors of the three ascoviruses and of the wasp *C. congregata*. However, they unambiguously indicate that there was at least one lateral transfer for this gene between the common ancestor of ascoviruses and the parasitic wasp.

Since iridoviruses, like ascoviruses and other virus species [44,45], are, in some cases, vectored by parasitic wasps, databases were mined using all the available ichnovirus virus proteins as queries. We found no significant relationships between CsIV, HfIV and TrIV genomes and genomes of their putative closest relatives NCLDV and non-NCLDV relatives. This indicates that passive lateral gene transfers from virus to eukaryotes that are successfully spread and maintained in ichnovirus genomes remain rare events. One case of such lateral transfer was described in the CcBV genome. In this genome, aside from the presence of cardinal endogenous eukaryotic retrotranposon and Polintons that transposed in the chromosomal DNA of the proviral form of CcBV [46-48], two genes encoding AcMNPV P94-related proteins, which have their closest relatives among granuloviruses (XcGV), were found. This suggests that CcBV contained at least two cases of lateral transfers between non-NCLDV and a bracovirus.

### Concluding remarks

Our results provide another source of evidence that passive lateral gene transfers have occurred regularly during evolution from bacteria to viruses and eukaryotes, and between viruses and eukaryotes [49-52]. Even if the pox-D5 NTPase genes in the GfIV genome, and the MCP and SfAV061-like genes in the CsIV genome, indicate that they have an ascovirus origin, they provide only limited evidence supporting an ascovirus origin of ichnoviruses. Indeed, their sequence conservation and biological characteristics suggest that there were repeated lateral transfers during evolution between ascoviruses and wasp genomes, including the proviral ichnovirus loci. This raises an important issue about the role of lateral transfers during co-evolution of the NCLDVs and non-NCLDVs, ichnovirus, wasp and parasitized host. Indeed, genetic materials of various origins have been exchanged and maintained during co-evolution. This therefore suggests that ichnoviruses might be chimeric entities partly resulting from several lateral transfer events of DNA fragments with viral origins.

Symbiogenesis was first proposed as an evolutionary mechanism when it became widely recognized that mitochondria and plastids originated from free-living prokaryotes [7]. The genomes of the endosymbiotic cyanobacteria and proteobacteria, respectively, at the origin of chloroplasts and mirochondria have evolved by reduction of several orders of magnitude to the approximate size of plasmids. Concurrently, nuclear genomes have been the recipients of plastid genomes. This relocation of the genes encoding most proteins of the endosymbiotic bacteria to the host nucleus is the ultimate step of this evolutionary process, so-called endosymbiogenesis [7,53]. Recent studies of plants have revealed a constant deluge of DNA from organelles to the nucleus since the origin of organelles [54]. This allows the host cell to have the genetic control on its organelles, in a relationship that is closer to enslavement or domestication than to a symbiosis or a mutualism in which the organelles would recover benefits from their contribution to the eukaryotic cell well-being. To date, this deluge of DNA is considered to correspond to passive lateral transfers that result from the interactions between the life cycle of the organelle and nuclear replication.

**Figure 5:**
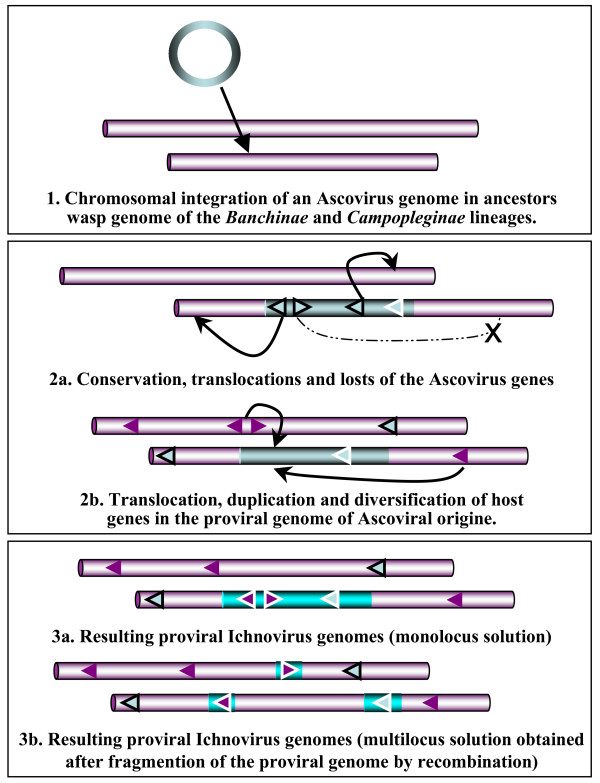
**Hypothetical mechanism for the integration and evolution of ascovirus genomes in endoparasitic wasps.** Schematic representation of the three-step process of symbiogenesis, and DNA rearrangements that putatively occurred in the germ line of the wasp ancestors in the *Banchinae *and *Campopleginae *lineages, from the integration of an ascoviral genome to the proviral ichnoviral genome. Sequences that originate from the ascovirus are in blue, those of the wasp host and its chromosomes are in pink. Genes of ascoviral origin are surrounded by a thin black or white line, depending on their final chromosomal location. Two solutions can account for the final chromosomal organisation of the proviral ichnovirus genome, monolocus or multilocus, since this question is not fully understood in either wasp lineage. More complex alternatives to this three-step process might also be proposed and would involve, for example, the complete *de novo *creation of a mono or multi locus proviral genome from the recruitment by recombination or transposition of ascoviral and host genes located elsewhere in the wasp chromosomes. This model for the chromosomal organization of proviral DNA in polydnaviruses is consistent with data recently published [58].

Numerous cases of symbiogenesis between endocellular bacteria and a wide variety of eukaryotic hosts have been characterized. However, recent work has demonstrated that this evolutionary process was not restricted to bacteria. It also occurred between endocellular eukaryotes such as unicellular algae and fungal endophyte in plants [55,56]. Endosymbiogenesis was also proposed as the evolutionary mechanism that allowed some invertebrate viruses with a large double-stranded DNA genome related to the nudiviruses and the ascoviruses [22], to have led, respectively, to the origin of bracoviruses and ichnoviruses, which are currently recognized as forming two genera within the family Polydnaviridae. Although presently there is no definitive evidence ruling out the hypothesis that the resemblance between ichnovirus and ascovirus virions is only an evolutionary convergence, the genomic differences between ascovirus and ichnoviruses are in good agreement with the symbiogenetic hypothesis. Indeed, they match an evolutionary scenario of endosymbiogenesis during which, from a single integration event of symbiotic virus genome, viral genes were lost and/or translocated from the provirus to other chromosomal regions (Fig. 5). In parallel, host genes of interest for the wasp parasitoid were integrated and diversified by selection and gene duplication in the proviral DNA. In this scenario, the more ancient symbiogenesis, the rarer the traces of genes from viral origin in the ichnovirus genome would be. This constitutes a constraint that dramatically limits the possibility to investigate the evolutionary links between ascovirus and ichnovirus. Results of our analyses demonstrate that the situation is also complicated by the fact that lateral gene transfers unrelated to the origin of ichnoviruses cause important misleading background noise. Moreover, the scenario in Figure 5 is close to a previously proposed version [57], but is not consistent with results presented here, nor with recently accumulated knowledge on DNA transfer from organelles into the nucleus. Since endocellular environments favour lateral transfers between virus and wasp nucleus, it can be proposed that genes of virus origin that are involved in the ichnovirus biology were passively integrated in one or several loci, step by step over time, alone or through transfers of gene clusters, or even the entire viral genome. Since parasitoid wasps are able to vector different viruses [44,45], this second scenario opens the exciting possibility that virus genes involved in the ichnovirus biology might correspond to a gene patchwork resulting from transfers from viruses belonging to different NCLDV and non-NCLVD families. Because of the background noise due to lateral gene transfers found in these systems, elucidating the origins of ichnoviruses will be very time-consuming, requiring new accurate experimental approaches to generate more robust evidence. Sequencing wasp genomes to identify proteins of viral origin that are components of virions and involved in the assembly of these may well contribute to our understanding of how ichnoviruses and bracoviruses evolved from other insect DNA viruses.

## Methods

### Database mining

Searches for similarities were mainly developed using facilities of BLAST programs at two websites http://www.ncbi.nlm.nih.gov/blast/Blast.cgi and http://genoweb.univ-rennes1.fr/Serveur-GPO/outils.php3?id_rubrique=47. For DpAV4 genes having their origin within eukaryotic, bacterial or virus genomes belonging to NCLDV and non-NCLDV families, the closest gene was located using the distance trees supplied with each BLAST search at the NCBI website.

### Defining syntactic model

Construction of syntactic models: Conserved amino acid blocks and positions described previously [15,22] and with new data sets were verified or determined using MEME at http://meme.sdsc.edu/meme/meme.html. In the first step, we used motifs resulting from MEME to make MAST minings in databases at http://meme.sdsc.edu/meme/mast.html. Since MEME motifs depend significantly on the data set use to calculate them, this approach did not enable an exhaustive detection of homologs among ascoviruses, iridoviruses, phycodnaviruses, mimiviruses and asfarviruses, and the detection sensitivity was ultimately very similar to that obtained with BLAST. To reach our detection objectives, we therefore constructed syntactic models that only included the most conserved positions and their variable spacing using WAPAM at the website. http://genoweb.univ-rennes1.fr/Serveur-GPO/outils_acces.php3?id_syndic=185&lang=en. Defining these models was obtained empirically until they allowed an exhaustive detection in refseq-protein and Genbank databases of the homologs among ascoviruses, iridoviruses, phycodnaviruses, mimiviruses and asfarviruses. The procedures were done until we were only able to detect exact match with the syntactic model. Whatever obtained with WAPAM, they required a confirmation with other approaches. Here, we used Psipred result comparison for regions with scores over 7 and HCA analyses for regions having scores lower than 7 with Psipred. This simplified the statistical treatment of the result obtained with WAPAM, since all exact matches have significance or a score of 100%.

Syntactic model for MCP orthologs in ascovirus and iridovirus genomes: G-[DE]-x(1)-ILMVFYW]-x(20,26)-[ILMVFYW]-x(5)-[ILMVFYW]-x(3)-[ILMVFYW]-[ILMVFYW]-x(4)- [ILMVFYW]-x(17)-[ILMVFYW]-x(10,16)-[ILMVFYW]-x(5,30)-[ILMVFYW]-x(1)-[ILMVFYW]-P- x(2)-[ILMVFYW]-[ILMVFYW]-[ILMVFYW]-x(3,6)-[AST]-x(14)-[ILMVFYW]-x(7)-[ILMVFYW]- [ILMVFYW]-[ILMVFYW]-x(18,70)-[ILMVFYW]-x(15)-P. The syntactic model for MCP orthologs in ascovirus, iridovirus, phycodnavirus and asfarvirus genomes: [GE]-[DE]-x(1)-[ILMVFYW]-x(20,80)-[ILMVFYW]-x(9)-[ILMVFYW]-[ILMVFYW]-x(4)-[ILMVFYW]- x(12,17)-[ILMVFYW]-x(10,16)-[ILMVFYW]-x(5,105)-[ILMVFYW]-x(1)-[ILMVFYW]- x(3)-[ILMVFYW]-[ILMVFYW]-[ILMVFYW]-x(3,6)-[AST]-x(14)- [ILMVFYW]-x(7)-[ILMVFYW]-[ILMVFYW]-x(18,90)-P.

Syntactic model for orthologs of the SfAV1a ORF061 in ascovirus, iridovirus, phycodnavirus and asfarvirus genomes: [FWY]-x(3)-[ILMVFYW]-[ILMVFYW]-x(3)-[RHK]-x(15,30)-[ILMVFYW]-x(2)-[ILMVFYW]-x(3)-LPC-x(2,4)-C-x(28,38)-[RHK]-x(2)-[ILMVFYW].

## Abbreviations

Acc N°: accession number; AcMNPV: *Autographa californica *multiple nucleopolyhedrovirus; BV: Bracovirus; CcBV: *Cotesia congragata *bracovirus; CIV: chilo iridescent virus; CsIV: *Campoletis sonorensis *Ichnovirus; DNA: deoxyribonucleic acid; DpAV4a: *Diadromus pulchellus *ascovirus 4a; GfIV: *Glypta fumiferanae *Ichnovirus; HfIV: *Hyposoter fugitivus *ichnovirus; HvAV3e: *Heliothis virescens *ascovirus 3e; IV: Ichnovirus; MCP: Major capsid protein; NCLDV: nucleocytoplasmic large DNA viruses; NTP: nucleotide tri-phosphate; ORF: Open reading frame; P: protein; RNA: ribonucleic acid; SfAV1a: *Spodoptera frugiperda *ascovirus 1a; TnAV2c: *Trichoplusia ni *ascovirus 2c; TrIV: *Tranosema rostrales *ichnovirus; VLP: virus-like particle; WAPAM: weighted automata pattern matching; XcGV: *Xestia c-nigrum *granulovirus.

## Authors' contributions

YB is the leader of all aspects of the research on the biology, genomics, and evolution of DpAV4. SS coordinated the sequencing, assembly, and sequence quality control of the DpAV4 genome. CAG participated in the bioinformatics analysis of the DpAV4 genome development of the manuscript. BAF contributed original concepts regarding the evolutionary origins and role of polydnaviruses in endoparasitoid biology, provided virological expertise to optimize data interpretation, and participated in writing the manuscript.

## Supplementary Material

Additional File 1 Predicted ORFs in DpAV4 genome. Table in which is indicated the ORF number, its location in the nucleic acid sequence, the size of the peptide encoded by each ORF and its Molecular mass, and its putative function. Click here for file

Additional File 2 Properties of the ORFs within the DpAV4 13 kbp regions containing homologs of the GfIV genes with a pox-D5 domain (Acc. N° CU467486). Table in which is described the properties of seven ORFs: ORF number, location within the 13-kbp of the Fig. 1., the size of the peptide encoded by each ORF and its Molecular mass, its pI, its putative function and its closest viral or cellular orhologs in databases. Click here for file

Additional File 3 Analysis of ascovirus and ichnovirus virion proteins. **3a: **Amino acid sequence alignment of the major capsid protein from selected large double-stranded DNA viruses. The seven conserved domains in MCP sequences of SfAV-1a, TnAV-2a, HvAV-3c, DpAV-4a, CIV, IV29, IV1, IV22, IV16, FV3, LCDV, CHV and ASFV were aligned. Identical and 100% similar positions are highlighted. **3b: HCA analysis of the conserved domain in MCP sequences**. For each of the seven conserved domain in MCP, clusters that are conserved in sequences and in HCA graphs are highlighted in yellow. **3c: **HCA analysis of the conserved domain in the MCP sequence of CsIV. Each of the seven conserved domain in MCP of ascoviruses, iridoviruses, phycodnaviruese and asfarvirus was located in the MCP sequence of CsIV. Clusters that are conserved in sequences and in HCA graphs are highlighted in yellow. **3d: **Amino acid sequence alignment of the protein encoded by orthologous genes of the SfAV1a ORF061. Sequences of SfAV1a ORF061 orthologs extracted from in DpAV4, HvAV3e, SfAV1a, TnAV2c, CIV, MIV, FV3, LCDV, SGV, PBCV, ESV and ASFV genome were aligned. Identical and 100% similar positions are highlighted. **3e: **HCA analysis of the conserved domain in SfAV1a ORF061. Clusters that are conserved in sequences and in HCA graphs of SfAV1a ORF061 orthologs are highlighted in yellow. Click here for file
