# Supplementary material for: Molecular evidence for the evolution of ichnoviruses from ascoviruses by symbiogenesis
Source: BMC Evol Biol. 2008 Sep 18;8:253. doi: 10.1186/1471-2148-8-253 (PMC2567993; doi:10.1186/1471-2148-8-253)
Supplement: Additional File 1 — Predicted ORFs in DpAV4 genome. Table in which is indicated the ORF number, its location in the nucleic acid sequence, the size of the peptide encoded by each ORF and its Molecular mass, and its putative function. [file 1471-2148-8-253-S1.doc]

**Lateral transfers between Polydnavirus and Ascoviruses**

Yves Bigot, Sylvie Samain, Corinne Augé-Gouillou and Brian A. Federici

Additional data file 1

**Predicted ORFs in DpAV4 genome (Acc N° : CU469068)**

| **ORF N°** | **Nucleotide positions in the genome** | **Peptide size (amino acids)** | **Molecular Mass** | **Putative Function** |
| --- | --- | --- | --- | --- |
| 1 | 1>2964 | 987 | 114.34 | DNA polymerase B |
| 2 | 2884<3363 | 159 | 18.03 | - |
| 3 | 3461>4234 | 257 | 29.17 | RNAseIII |
| 4 | 4304>5236 | 310 | 35.75 | BRO-like protein 1 |
| 5 | 5274<5760 | 161 | 18.59 | NUDIX hydrolase |
| 6 | 5842>6240 | 132 | 15.01 | - |
| 7 | 6273>6887 | 204 | 23.22 | IAP-like protein |
| 8 | 6927>8168 | 413 | 44.48 | Cofactor of the virion S/T kinase encoded by the DpAV4 ORF044 |
| 9 | 8213>9004 | 263 | 29.46 | - |
| 10 | 9166>9654 | 162 | 18.56 | RNA polymerase II, subunit-like Rpb5b |
| 11 | 9721>10185 | 154 | 18.4 | - |
| 12 | 10186>11232 | 348 | 39.27 | - |
| 13 | 11268<11990 | 240 | 27.45 | - |
| 14 | 12278<12574 | 98 | 11.62 | - |
| 15 | 12613>13392 | 259 | 30.01 | Fatty acid elongase |
| 16 | 13415<14371 | 318 | 36.78 | BRO-like protein 2 |
| 17 | 14415<15404 | 329 | 37.75 | BRO-like protein 3 |
| 18 | 15450<15812 | 120 | 14.03 | - |
| 19 | 15955>17259 | 434 | 49.92 | Major Capsid Protein |
| 20 | 17284<19851 | 855 | 96.06 | SNF2 DEAD-like helicase |
| 21 | 19869>20546 | 225 | 25.50 | - |
| 22 | 20575>20886 | 103 | 12.32 | Yabby-like transcription factor |
| 23 | 20929>21522 | 197 | 20.30 | - |
| 24 | 21554>22087 | 177 | 19.91 | - |
| 25 | 22147>22962 | 271 | 31.07 | Polymerase processivity factor for DNA replication & repair |
| 26 | 22977>24014 | 345 | 39.29 | Metallo-dependent calcineurin-like phosphatase |
| 27 | 24042<25664 | 540 | 60.02 | - |
| 28 | 25718>26290 | 190 | 22.50 | Uvr/REP helicase |
| 29 | 26294>26965 | 223 | 24.95 | Zinc-dependent metalloprotease |
| 30 | 26730<27001 | 89 | 9.82 | - |
| 31 | 27333>27980 | 215 | 24.98 | - |
| 32 | 28023<28985 | 320 | 36.95 | BRO-like protein 4 |
| **ORF N°** | **Nucleotide positions in the genome** | **Peptide size (amino acids)** | **Molecular Mass** | **Putative Function** |
| 33 | 29026<30918 | 630 | 71.53 | SbcC subunit C |
| 34 | 31222<32283 | 353 | 40.90 | BRO-like protein 5 |
| 35 | 32311<33153 | 280 | 31.61 | - |
| 36 | 33388>34941 | 517 | 58.82 | Serine/threonine protein kinase |
| 37 | 34866>35513 | 215 | 25.26 | - |
| 38 | 35544<35963 | 139 | 15.29 | - |
| 39 | 35960<36448 | 162 | 18.49 | - |
| 40 | 36470<37801 | 443 | 47.24 | - |
| 41 | 37816>38130 | 104 | 12.10 | Evrl/Alr thiol oxidase |
| 42 | 38140>38520 | 126 | 15.06 | - |
| 43 | 38553>39638 | 361 | 41.30 | - |
| 44 | 39872>40267 | 131 | 18.89 | Thiredoxin |
| 45 | 40273>40620 | 115 | 13.36 | - |
| 46 | 40642<43065 | 807 | 90.15 | Serine/threonine protein kinase |
| 47 | 43099<43544 | 148 | 16.61 | BRO-like protein 6 |
| 48 | 43561<44754 | 397 | 44.05 | Cathepsin B |
| 49 | 44744<45238 | 164 | 18.29 | - |
| 50 | 45278>45655 | 125 | 14.27 | BRO-like protein 7 |
| 51 | 45692<46354 | 220 | 24.82 | - |
| 52 | 46801<47325 | 174 | 19.86 | ABC-type transport system permease |
| 53 | 47353<47910 | 185 | 19.93 | - |
| 54 | 47933<49672 | 580 | 65.53 | Ribonucleotide reductase |
| 55 | 49829>50392 | 187 | 22.01 | Thymidine kinase |
| 56 | 50778>51140 | 120 | 13.82 | metallo-hydrolase |
| 57 | 51447>51749 | 100 | 11.40 | metallo-hydrolase |
| 58 | 52415<52774 | 119 | 13.58 | DNA-directed RNA polymerases I, II, and III subunit RPABC2 |
| 59 | 53193>53654 | 153 | 17.77 | Acyl-Coenzyme A Binding Protein |
| 60 | 54045>54692 | 215 | 25.27 | - |
| 61 | 54735<55253 | 172 | 18.64 | ABC-type transport system permease |
|  | 55460-55550 |  |  | miRNA ORF-like |
| 62 | 55254<55502 | 82 | 9.73 | Ubiquitin |
| 63 | 55573>56133 | 186 | 21.52 | - |
| 64 | 56178>57362 | 394 | 44.67 | - |
| 65 | 57359<58204 | 281 | 31.58 | Myristylated membrane protein-like |
| **ORF N°** | **Nucleotide positions in the genome** | **Peptide size (amino acids)** | **Molecular Mass** | **Putative Function** |
| 66 | 58252>59439 | 395 | 46.09 | Ribonucleotide reductase |
| 67 | 59514>60504 | 329 | 37.73 | BRO-like protein 8 |
| 68 | 60703>61965 | 420 | 48.54 | NTPase/helicase |
| 69 | 62343<62819 | 158 | 18.29 | - |
| 70 | 62850<64121 | 423 | 47.63 | DNA-directed RNA polymerase subunit 1 |
| 71 | 64723>65112 | 129 | 15.26 | - |
| 72 | 65139>65501 | 120 | 14.41 | - |
| 73 | 65537>68641 | 1034 | 115.23 | DNA-directed RNA polymerase subunit 2 |
| 74 | 68680<69085 | 134 | 16.14 | - |
| 75 | 69115<70410 | 431 | 50.14 | BRO-like protein 9 |
| 76 | 70740>71330 | 196 | 22.59 | - |
| 77 | 71774>72043 | 89 | 10.07 | Endonuclease |
| 78 | 72165>73112 | 315 | 37.18 | - |
| 79 | 73153<74463 | 436 | 50.60 | - |
| 80 | 74463<75575 | 370 | 41.86 | Serine/Threonine protein kinase |
| 81 | 75578<76432 | 284 | 32.54 | PlsC phosphate acyltransferase |
| 82 | 76537>76911 | 124 | 13.95 | Transcription elongation factor-SII |
| 83 | 77466<78239 | 257 | 28.51 | Hydroxysteroid (17-beta) dehydrogenase |
| 84 | 78364>78837 | 157 | 17.54 | - |
| 85 | 78876>82001 | 1041 | 117.91 | Dynein-like beta chain |
| 86 | 82016<82906 | 296 | 34.50 | ATPase3 |
| 87 | 82929<83738 | 269 | 30.36 | Patatin-like phospholipase |
| 88 | 83790>84209 | 139 | 15.79 | - |
| 89 | 84276>86897 | 873 | 98.74 | DNA-directed RNA polymerase subunit alpha |
| 90 | 87212>89989 | 925 | 105.35 | DNA primase with a pox D5 ATPase domain |
| 91 | 90251>90736 | 162 | 19.12 | - |
| 92 | 91296>91919 | 207 | 23.94 | ALI-like protein |
| 93 | 92072>94621 | 849 | 97.31 | Putative ATPase |

| **ORF N°** | **Nucleotide positions in the genome** | **Peptide size (amino acids)** | **Molecular Mass** | **Putative Function** |
| --- | --- | --- | --- | --- |
| 94 | 94677>96914 | 745 | 84.67 | Putative chromosomal replication initiation protein |
| 95 | 96959>97450 | 163 | 18.72 | Zinc finger protein |
| 96 | 97525>98874 | 449 | 51.19 | E3 ubiquitin ligase |
| 97 | 98901>99359 | 152 | 17.65 | - |
| 98 | 99515>100726 | 403 | 46.33 | BRO-like protein 10 |
| 99 | 100731<100949 | 72 | 8.32 | - |
| 100 | 101067<101342 | 91 | 10.86 | - |
| 101 | 101369<101740 | 123 | 14.28 | - |
| 102 | 101764<102407 | 213 | 20.06 | - |
| 103 | 102396<103509 | 370 | 43.18 | Late Transcription Factor VLTF3-like |
| 104 | 103879<103499 | 128 | 14.44 | Putative Thioredoxin |
| 105 | 104077<106494 | 805 | 91.15 | Protein kinase |
| 106 | 106577>106819 | 80 | 9.11 | - |
| 107 | 106849>107268 | 139 | 15.74 | - |
| 108 | 107348>107815 | 155 | 18.08 | Zn-finger/nucleic acid binding protein |
| 109 | 108205>109080 | 291 | 33.08 | Delta 9 fatty acid desaturase |
| 110 | 109300>110331 | 343 | 39.62 | BRO-like protein 11 |
| 111 | 110593>111462 | 289 | 30.96 | Oxidoreductase |
| 112 | 111429>112526 | 365 | 42.41 | BRO-like protein 12 |
| 113 | 114516<114010 | 164 | 19.94 | Putative DNA ligase |
| 114 | 114060<114515 | 151 | 16.23 | - |
| 115 | 114533<115576 | 347 | 39.37 | CK1 family protein kinase |
| 116 | 115674>116244 | 189 | 20.05 | - |
| 117 | 116273<116803 | 176 | 20.54 | CDT-like phosphatases |
| 118 | 116838>117911 | 357 | 40.39 | Metallo-hydrolase |
| 119 | 117947<119131 | 394 | 44.14 | RedQ-like DEAD helicase |
