# Supplementary material for: Molecular evidence for the evolution of ichnoviruses from ascoviruses by symbiogenesis
Source: BMC Evol Biol. 2008 Sep 18;8:253. doi: 10.1186/1471-2148-8-253 (PMC2567993; doi:10.1186/1471-2148-8-253)
Supplement: Additional File 2 — Properties of the ORFs within the DpAV4 13 kbp regions containing homologs of the GfIV genes with a pox-D5 domain (Acc. N° CU467486). Table in which is described the properties of seven ORFs: ORF number, location within the 13-kbp of the Fig. 1., the size of the peptide encoded by each ORF and its Molecular mass, its pI, its putative function and its closest viral or cellular orhologs in databases. [file 1471-2148-8-253-S2.doc]

**Lateral transfers between Polydnavirus and Ascoviruses**

Yves Bigot, Sylvie Samain, Corinne Augé-Gouillou and Brian A. Federici

Additional data file 2

**Properties of the ORFs within the DpAV4 13kbp regions containing homologs of the GfIV genes with a pox-D5 domain (Acc. N° CU467486).**

| ORF | Nucleotide position in the fragment | N° of amino acids | Theoretical  MW (Da) | Theoretical  pI | Putative function of encoded peptide | Closest viral or cellular orthologs |
| --- | --- | --- | --- | --- | --- | --- |
| 89 | 31>2649 | 873 | 98744 | 7.90 | RNA polymerase 1 subunit C | CIV ORF176R  MIV ORF090L  TnAV2c ORF 042  HvAV3e ORF 011  SfAV1a ORF008  LCDV ORF116L |
| 90 | 2967>5741 | 925 | 105355 | 6.54 | Putative DNA-primase | GfV-C20-ORF1  GfV-D4-ORF1  GfV-D1-ORF1 |
| 91 | 6003>6488 | 162 | 19125 | 8.49 | Unknown function | GfV-D1-ORF1  GfV-D4-ORF1  GfV-D3-ORF2 |
| 92 | 6661>7281 | 207 | 23945 | 5.93 | ALI-like protein | CIV ORF069L  MSV ORF196  MSV ORF024  MSV ORF026 |
| 93 | 7437>9983 | 849 | 97315 | 7.12 | Putative ATPase | GfV-C20-ORF1  GfV-C21-ORF1  GfV-D4-ORF1  GfV-D1-ORF1  GfV-D2-ORF1  GfV-D3-ORF2  CIV ORF184R  MIV ORF121R  HvAV3e ORF119  SfAV1a ORF099  TnAV2c ORF078  LCDV ORF078  (Chinese isolate) |
| 94 | 10042<12276 | 745 | 84673 | 4.9 | Putative chromosomal replication initiation protein | MIV ORF113L  CIV ORF155L |
| 95 | 12234>12812 | 163 | 18728 | 9.14 | Zinc finger protein | CIV ORF132L |

MSV = Melanoplus sanguinipes entomopoxvirus
