# Supplementary material for: Molecular evidence for the evolution of ichnoviruses from ascoviruses by symbiogenesis
Source: BMC Evol Biol. 2008 Sep 18;8:253. doi: 10.1186/1471-2148-8-253 (PMC2567993; doi:10.1186/1471-2148-8-253)

**Lateral transfers between Polydnavirus and Ascoviruses**

Yves Bigot, Sylvie Samain, Corinne Augé-Gouillou and Brian A. Federici

Additional data file 3

Figure 3a

| **Domain 1**  50 60 70 80 90 100 | **Domain 2** | |
| --- | --- | --- |
|  | 190 200 210 220 230 240 | |
| SfAV-1a ID--NYIYGPDERVTTYFVREIRPCAAFFKLPVLLTNGNGMKKFGGTFTMPINAS-GDYL … HKLIKSVKLKIDGKPLVELSSSFMDMWSEFMIDNGNYEAYNNMVG---GEYE- | |  |
| TnAV-2a ID--SYIYGPDEQVTTYFVREIRPCAAFSKMPVLLNTGNGSNKFGGTYTMPINAS-EDYL … HKLIKSIRLKVDAKPLVELSSNFMDTWSEFMIDGGNYDAYTNMVG---GDFR- | |  |
| HvAV-3c ID--SYIYGPDEQVTTYFVREIRPCAAFSKMPVLLNTGNGSNKFGGTYTMPINAS-GDYL … HKLIKSIRLKVDAKPLVELSSNFMDTWSEFMIDGGNYDAYTNMVG---GDFQ- | |  |
| DpAV-4a LE--NHLYDTS-SAYSYFKRDYKKTSPVFKTPVVLDKTTGTSSFSYDWSAFVDKTQGDYL … HNLLEECTLTFNDTVVNRLDSCILDFISEFSVDESKYSQYMKNIGNQQCLLQ- | |  |
| CIV IE--KYMYGGK-TSTAYFVRETRKATWFTQVPVSLTRANGSANFGSEWSASISRA-GDYL … HNLIRECSITFNDLVAARFDHYHLDFWAAFTTPASKAVGYDNMIGNVSALIQP | |  |
| IV29 IE--KYMYGGP-TATAYFVREIRKSTWFTQVPVPLSRNTGNAAFGQEWSVSISRA-GDYL … HNLIREATITFNDLVAARFDNYHLDFWSAFTVPASKRNGYDNMIGNVSSLIN- | |  |
| IV1 IE--KYMYGGP-TATAYFVREIRKSTWFTQVPVPLSRNTGNAAFGQEWSVSISRA-GDYL … HNLIREATITFNDLVAARFDNYHLDFWSAFTVPASKRTGYDNMIGNVSSLIN- | |  |
| IV22 IE--KYMYGGP-TATAYFVREIRKSTWFTQVPVPLSRNTGNAAFGQEWSVSISRA-GDYL … HNLIREATITFNDLVAARFDNYHLDFWSAFTVPASKRNGYDNMIGNVSSLIN- | |  |
| IV16 IE--KYMYGGP-TATAYFVREIRKSTWFTQVPVPLSRNTGNAAFGQEWSVSISRA-GDYL … HNLIREATITFNDLVAARFDNYHLDFWSAFTVPASKKIGYDNMIGNISALTN- | |  |
| FV3 LE--RAMYGGS-DATTYFVKEHYPVGWFTKLPSLAAKMSGNPAFGQQFSVGVPRS-GDYI … HNIVESVTLSFNDISAQSFNTAYLDAWSEYTMPEAKRTGYYNMIGNTSDLINP | |  |
| LCDV IE--KHLYGGD-SAVAYFVRETKKCTWFSKLPVLLTRCSGSPNFDQEFSVNVSRG-GDYV … HNLIKQTSVQFNDLVAQKFESYFLDYWAAFSMCGSKRAGYNNMIGNTIDMIQP | |  |
| CHV QD--VYLTGNP-QIT--FFKTVYRRYTNFAIESIQQTINGSVGFGNKVSTQISRN-GDLI … --LLQDVELEIGGQRID---KHYNDWFRTYDALFRMNDDRYNYRRMT-DWVN- | |  |
| ASFV PDPEPTLSQIEETHMVHFNAHFKPYVPIGFEYNKVRPHTGTPTLGNKLTFGIPQY-GDFF … ERLYENVRFDVNGNSLDEYSSDVTTLVRKFCIPGDKMTGYKHLVGQEVSVEGT | |  |

| **Domain 3**  **Domain 4 Domain 5** 340 350 360 370 460 470 480 500 510 |
| --- |
| SfAV-1a -----KSVVLPIPMYFSRDTGIALPIGAMVNNRVTVEFVRRKVTDLLVLE … TSCTPHYMYMEKPVDVPATEL-VKPTD-GTP--APDTMSFEIEHTNGIVKALFFGVRNITRP |
| TnAV-2a -----KTLVLPIPLYFSRDSGVALPIGMMVNNRVTVEFVFRKLSDLLIKE … TSCSPHYILMEKPTDIAATEI-LKPTE------AGSTLSYDIEHSSGILKALFFGVRNITHS |
| HvAV-3c -----KTLVLPIPLYFSRDSGVALPIGMMVNNRVTVEFVFRKLSDLLIKE … TSCSPHYILMEKPTDIAATEI-LKPTE------AGSILSYDIGHSSGILKALFFGVRNITHS |
| DpAV-4a ----SRCMVIPIPFFFNESVRNALPLSEMPYTEIKINFKFRAWESLVLLE … VSEEERRTIGVRPSTMVFKQYQILPRQKV----TDEKTKIQLMFKHS-VTKIYFGIRNSTFK |
| CIV -ATVSADLNLPLPFFFSRDSGVALPTAALPYNEMRINFQFHDWQRLLILD … VSNEERRRMGCSVRDILVEQVQTAPRHVWNP-TTNDAPNYDIRFSHA-IKALFFAVRNTTFS |
| IV29 GSVGGINLNLPLPFFFSRDTGVALPTAALPYNEMQINFNFRDWHELLILT … VSNEERRRMGCAIRDILIEQVQTAPRQNYVP-LTNASPTFDIRFSHA-IKALFFAVRNKTSA |
| IV1 GSTGGTNLNLPLPFFFSRDTGVALPTAALPYNEMQINFNFRDWTELLVLQ … VSNEERRRMGCAIRDILIEQVQTAPRQNYTP-LTNASPTFDIRFSHA-IKALFFSVRNKTSA |
| IV22 GSVGGINLNLPLPFFFSRDTGVALPTAALPYNEMQINFNFRDWHELLILT … VSNEERRRMGCAIRDILIEQVQTAPRQNYVP-LTNASPTFDIRFSHA-IKALFFAVRNKTSA |
| IV16 GSVGGINLNLPLPFFFSRDTGVALPTAALPYNEMQINFNFRDWPELLILT … VSNEERRRMGCAIRDILIEQVQTAPRQNYTP-LTNAMPTFDIRFSHA-IKALFFSVRNKTSS |
| FV3 RVLPAKNLVLPLPFFFSRDSGLALPVVSLPYNEIRITVKLRAIHDLLILQ … ITGDERQAMSSTVRDMVVEQVQAAPVHVVNP-RNATTFHTDMRFSHA-VKALMFMVQNVTHP |
| LCDV GMLPEKVLVLPLPYFFSRDSGVALPSAALPYNEIRLTFHLRDYTELLIFQ … VTNEERRLMGTTPRDILVEQVQTAPKHVFQP-LTIPSPNFDIRFSHA-IKLLFFGVRNTTHA |
| CHV GAQ--KRFYVPLIFFFNQTPGLALPLIALQYHEVKLYFTLASQVQGVNYN … LDTQERTRFAQLPHEYLIEQLQFTGSETATP-SATTQASQNIRLNFN-HPTKYLA-WNFNNP |
| ASFV YYQPPLALWIKLRFWFNENVNLAIPSVSIPFGERFITIKLASQKDLVNEF … HHDEKLMSALKWPIEYM-----FIGLKPTWNISDQNPHQHRDWHKFG-HVVNAIM--QPSHH |

##

| Domain 6 Domain 7 590 600 610 620 630 670 680 700 |
| --- |
| SfAV-1a ---SEHFVYTEP-YHAAKRVPTTNNGLYMYSFALDL-HTIDPKGSINPSNFNSNISV … TERFEFN----ATVLTGYILYVENGNLRKIDNGGDFN…… |
| TnAV-2a ---AEHFVFTEP-YHAACRVPTRNRGQYMYSFALDL-KTVDPKGSINPPNFNSSISV … AETFEFT----AIALTSYILYIDNGSLKKIDNGGDFN…… |
| HvAV-3c ---AEHFVFTEP-YHAACRVPTRNRGQYMYSFALDL-KTVDPKGSINPSNFNSSISV … AETFEFT----AIALTSYILYIDNGSLKKIDNGGDFN…… |
| DpAV-4a ---DSYFKMIEPIYCNR-RAPEKE-GHFVYNFDIMS-DSPYASGSLAVSRLNNPTME … DRQFEFI----TIAENTNVITISEGNAQVPVLH----…… |
| CIV ---SDYFSLVNPWYHAP-TIPGLT-GFHEYSYSLAF-NEIDPMGSTNYGKLTN-ISI … PQTFEFI----VTALNNNIIRISGGALGFPVL-----…… |
| IV29 ---SDYFSLINPFYHAP-TIPSFI-GYHLYSYSLHF-YDLDPMGSTNYGKLTN-VSV … PQSYEFV----ILAVNNNIVRISGGETPQNYIAVC--…… |
| IV1 ---SDYFSLINPFYHAP-TIPSFI-GYHLYSYSLHF-YDLDPMGSTNYGKLTN-VSV … PQNYEFV----IVAVNNNIVRISGGETPQNYLSGSFVTL |
| IV22 ---SDYFSLINPFYHAP-TIPSFI-GYHLYSYSLHF-YDLDPMGSTNYGKLTN-VFV … AQSYEFV----IVAVNNNIVRIENSLVRNRRRWSREGPM |
| IV16 ---SDYFSLINPFYHAP-TIPSSI-GYHLYSYSLHF-FDLDPMGSTNYGKLTN-VSV … AQSYEFV----IIGVNNNIIRISGGALGFPVL-----…… |
| FV3 ---VDYYSLVEPWYYAT-SIPVST-GHHLYSYALSL-QDPHPSGSTNYGRLTN-ASL … AQKYALI----VLAINHNIIRIMNGSMGFPIL-----…… |
| LCDV ---SEYYSLVQPYYFGG-SIPIET-GYHMYCYSLNM-MDMDPMGSTNYGRLSN-VSM … AQKFEFL----TMAINHNVIRIKNGSMGFPVL-----…… |
| CHV TRKGSYFNKVQPYQSIG-GVTPA--GVYLYSFALKP-AGRQPSGTCNFSRIDN-ATL … ANTATLLTALNIYAKNYNVLRIMSGMGGLAYAN----…… |
| ASFV SKFCSSYIPFHIGGNSI--KTPSSPGAMMITFALKPREEYQPSGHINVSRARE-FYI … ASAINFLLLQNGSA----VLRYST-------------…… |

**Amino acid sequence alignment of the major capsid protein from selected large double-stranded DNA viruses**. Identical and 100% similar positions are highlighted in black boxes. Positions conserved in 11 of the 12 sequences or in members of at least 3 virus families are highlighted in gray boxes. The matrix of substitutions used was that described by Nevill-Manning *et al*.,1998. Only one substitution group was added: R=K=H. (1) SfAV-1a, (2) TnAV-2a, (3) HvAV-3c, (4) DpAV-4a, (5) CIV, (6) IV29, (7) IV1, (8) IV22, (9) IV16, (10) FV3, (11) LCDV, (12) CHV and (13) ASFV.

Figure 3b

**HCA analysis of the conserved domain in MCP sequences.** For each domain, clusters that are conserved in sequences and in HCA graphs are highlighted in yellow.

**
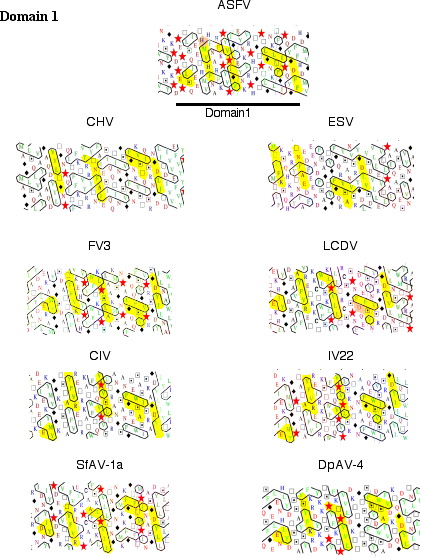
**

**
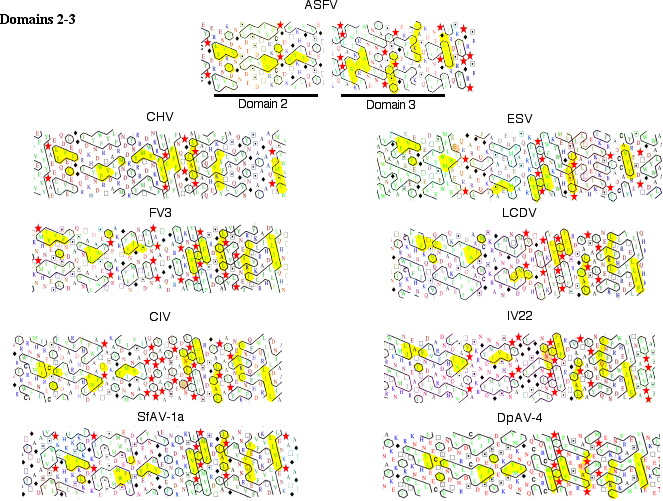
**

**
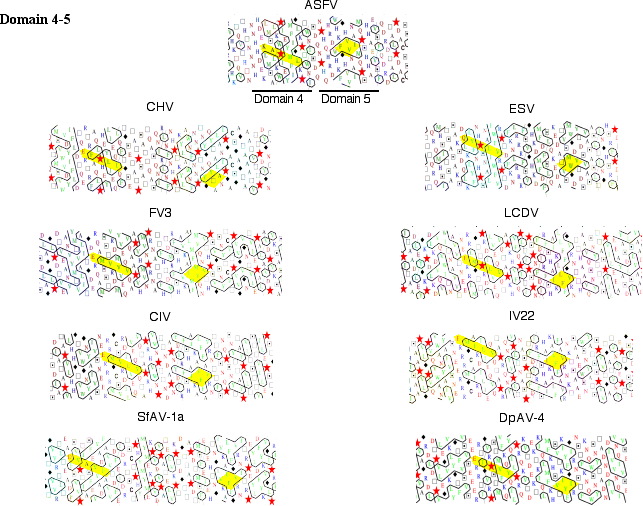
**

**Figure 3c**

HCA analysis of the conserved domain in the MCP sequence of CsIV. For each domain, clusters that are conserved in sequences and in HCA graphs with those of their ascovirus, iridovirus and phycodnavirus relatives are highlighted in yellow.

**
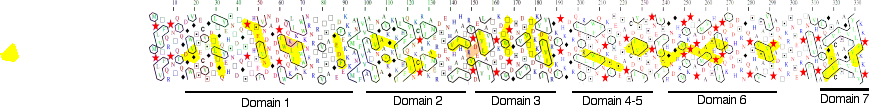
**

**Amino acid sequence of the CsIV MCP:**

MPLVTFQRPNTQIQGDGWVGSSECVSGDTLTGQYEHSNTDTQGSPTTHSEFVNDNEAGDD

PIWQQKLIKIWWKNRNRTRTVTSATTGEDFEISKDDKEEHSAISSYVRLKKKMANLFVVA

ATIVNGAAVKGKGKIIHFQVLNKCPRYIKAMEAAFRDSSENHVHAFHATLRYPDEYVLAV

ITSPKDTWTPDGSNVDSVTTDKKYDIVIQRGKFPHKGDGRQLEIRLSSWFNQFNPKSISE

SESAPSCDIVSQVSSSPNEYVSLHGNDGHRMPVKPTAPAMDAVQSHPIIPQKLDDHDSRN

GQPSHAVSATVYSPADNTSKRSNSETRPTNFIDFAVHPALQIGSVCALSAGVTYLLVKKP

KLAS

**Figure 3d**

DpAV4 ------------------------MSKPIPHPSVWGPHFWYVIHTYSDTY-PEQPNVVDVEVA------------SQFIKILPFLLPCNE

HvAV3e --------------MFLKANNSF--KKNYNGPEWWGPHYWYFLH-WHADL-NVSHGDARSRNEV-----------AWLLRNVHSVLPCAS

SfAV1 --------------MFSRSNRTSSPSRHPNGPDVWGPHYFLHWH---ADV-STTTFV--NRTSQ----------IAW-LRNLHMVLPCPS

TnAV2c ---------MNVKKITEVLVNLEPGPLFVGGPDVWGPHFWFLLH-WFSDKYLSSKQFHAQSYHD----------AAWLIRNIHAVIPCIT

CIV --------------------------MTDIDPHIWGPSFWSTYHLYASSYPIHPTPI-IMDAAR------------SFVKTIPFTLPCSS

MIV ---------------------------MSIDPKLWGNAFWSTLHHVAAGYNDHPSLGARQVMTN-------------FIQSIPVLLPCAE

FV3 ----MHGCNCNRVSGHLSAVRSSGLENGPFGPSGFGPSMWFTMHSGAAERAIRGGYLTENEKVA----------WESWLRNLWVCIPCES

LCDV -----MNRYLKKSQTQEVVVMHSKPTQNAFNPISFGPSLWYSLHTAASSI-SDPITV--TDKKD----------WVNLLKSLAVLLPCHA

SGV MCLSIKMQRRYSVESKLSVKNASLLEGGGFGPHGFGPAMWFTFHTGAAAQACKGGSLTASEHEA----------WEHFIRSMWVCIPCQT

PBCV --------------------MNSTNNVPNFDPNIWGPSVWLMIHLSALRYPKNPTAV---DKKN----------FAAFYRSFPFILPCTG

ESV ------------------mnnekmndnnglctstwgppgwfflhcvaagypvdpdey---ddirgntrghtrrgyssffkntghvlpcrf

ASFV -------------------------------MLHWGPKYWRALHLYAIFFSDTPGWK---EKYE----------AIQWILNFIESLPCTR

DpAV4 CAKHAFDYIKPFVEDEQVLKNIVKNKESLSAFFHNFHNAVNIRTGKPVFHK---------------------------------------

HvAV3e CAHEAYTYSSSHRVQFP---HVVHNPELNVIYWRDMHNAVNRRLGKPIVVIAP--------VRRDYSFATWWSHCSVVVTACCEDFGSRS

SfAV1 CAVEAYEYSSKRLEQFP---YIVND-ARAYVSWRNMHNHVNRRLGKPLMIIEP--------LRSDYSLSTWWYHCSIVIRTCIEDFA---

TnAV2c CAQEAYEYGQKHLLLFR---QITANPMEYALYWFRFHNFVNRRLNKMIKVKFT-------RFESPPELSVWWEHCDIVIRSSGEDNSTRL

CIV CTDHAFAYIKNIQKQDPDLISIVSSKMLFEKFFIDFHNSVNYRLGKPLL---------------PESVARKKWRF---------------

MIV CQDHAFDYIGRADLD-----RVVSSRRQLFLFFFNFHNHVNARLNKPQL--------------AAKTVFQRYRVPFDGEAAAATTEPPFH

FV3 CRRHYMGIVNAVDF------GSVNTGDKIFRLTVDIHNMVNARLNKPH-----------------VTLQKAIHIYGLDTKLGPASTITFR

LCDV CKQHYTDIIKRTDLN-----QVTNTKKQLFCFLVDVHNVINLRTNKPE-----------------FSYNKAKKLYGYNG--GPNLMLFIN

SGV CSVHFKNMLRDIDF------TLVCTGIDVFKLSVDMHNRVNTRLNKPC-----------------MSLEKAKRLYGLDTKTGPPVVVSYR

PBCV CCKGFTKILEMTKFGA----KDLQSRDTLFAWTVKAHSLVNIKTGKPP--------------RDEPEY----------------------

ESV crdsyvhfssetpvee----yl-hsrqalfewlfiihnkvndkigdkqet------------dlesvvdkyerfrakchhqkatgctdpa

ASFV CQHHAFSYLTKNPL------TLNNSEDFQY-WTFAFHNNVNNRLNKKIISWSEYKNIYEQSILKTIEYGKTDFIGAWSSL----------

DpAV4 ---------------------------------------------------------------------------- Ascovirus

HvAV3e DSLDTCLTFIDIVLDSYRMMVDVNLSESVAVIEAKRNLFTNIRTNGRLRAMRKFCADIFDIVQHAASS-------- Ascovirus

SfAV1 ---------------------------------------------------------------------------- Ascovirus

TnAV2c DNVKFFVDFVELIIRACPQYSLWRVMEHLLVLERNNLFDKLQYENNEHQRSILIKKYTIKICDTIDNYIKIRQYKS Ascovirus

CIV ---------------------------------------------------------------------------- Iridovirus

MIV WSPWLTTALAVILVVVVAGIGHRSRFK------------------------------------------------- Iridovirus

FV3 ANTSTFNIILDSHPSLRSSIVYHPYVVEARTRLSMEIGTPSSRREAFVDYYNAIETIVMQYVNEYQRVR------- Iridovirus

LCDV PDVNIFD--------------------------------------------------------------------- Iridovirus

SGV ARLFQ----------------------------------------------------------------------- Iridovirus

PBCV ---------------------------------------------------------------------------- Phycodnavirus

ESV rhnekmicrlvvepvnrkrnrwaalsimavliifvlfy-------------------------------------- Phycodnavirus

ASFV ---------------------------------------------------------------------------- Asfarvirus

**Amino acid sequence alignment of the protein encoded by orthologous genes of the SfAV1a ORF061**. Identical and similar positions are highlighted in black boxes. The matrix of substitutions used was that described by Nevill-Manning *et al*.,1998. Only one substitution group was added: R=K=H. DpAV4 ortholog is encoded by the minus strand of the sequence referenced with the Acc. N° ADP279815

**Amino acid sequence of one of three CsIV ortholog (M80623) of the SfAV1a ORF O61:**

MPKSDCINWVARVLICSRRKRTSCQLLTYQLWYSSCRTVASLAFIKRLPCQKRLCFKCLA

AVISSIATCARHQPYQSYLALIMTHSNYIIYDKNVCSEQMTWCRCRS

**Other CsIV sequences are referenced:** S47226, AF236017, AF362508

**Figure 3e**

**HCA analysis of the conserved domain in SfAV1a ORF061**. For each domain, clusters that are conserved in sequences and in HCA graphs are highlighted in yellow.


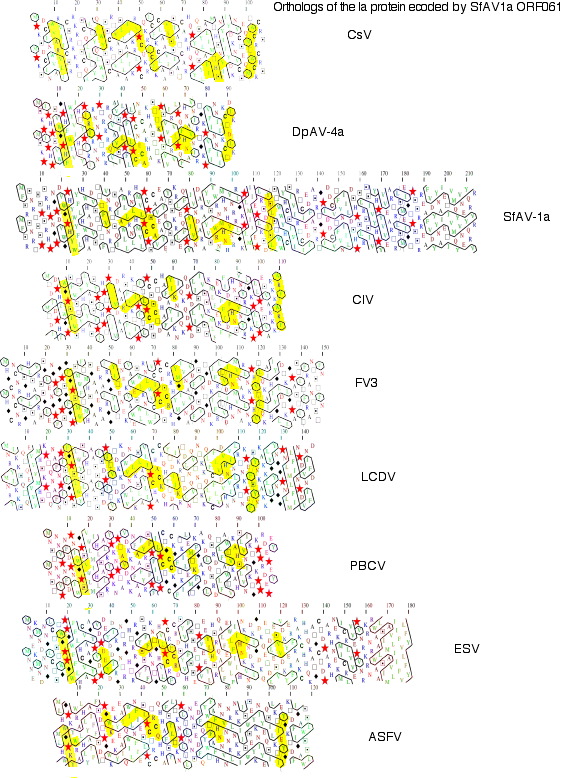

Supplement: Additional File 3 — Analysis of ascovirus and ichnovirus virion proteins. 3a: Amino acid sequence alignment of the major capsid protein from selected large double-stranded DNA viruses. The seven conserved domains in MCP sequences of SfAV-1a, TnAV-2a, HvAV-3c, DpAV-4a, CIV, IV29, IV1, IV22, IV16, FV3, LCDV, CHV and ASFV were aligned. Identical and 100% similar positions are highlighted. 3b: HCA analysis of the conserved domain in MCP sequences. For each of the seven conserved domain in MCP, clusters that are conserved in sequences and in HCA graphs are highlighted in yellow. 3c: HCA analysis of the conserved domain in the MCP sequence of CsIV. Each of the seven conserved domain in MCP of ascoviruses, iridoviruses, phycodnaviruese and asfarvirus was located in the MCP sequence of CsIV. Clusters that are conserved in sequences and in HCA graphs are highlighted in yellow. 3d: Amino acid sequence alignment of the protein encoded by orthologous genes of the SfAV1a ORF061. Sequences of SfAV1a ORF061 orthologs extracted from in DpAV4, HvAV3e, SfAV1a, TnAV2c, CIV, MIV, FV3, LCDV, SGV, PBCV, ESV and ASFV genome were aligned. Identical and 100% similar positions are highlighted. 3e: HCA analysis of the conserved domain in SfAV1a ORF061. Clusters that are conserved in sequences and in HCA graphs of SfAV1a ORF061 orthologs are highlighted in yellow. [file 1471-2148-8-253-S3.doc]
